# Case report: An unusual case of small bowel bleeding and common iliac artery pseudoaneurysm caused by an unnoticed swallowed toothpick

**DOI:** 10.3389/fmed.2023.1182746

**Published:** 2023-06-08

**Authors:** Yao Xiong, Jing Yan, Gaowu Yan, Lei Feng, Yong Li, Suyu He, Ruyi Li, Gangcheng Tan, Bo Feng

**Affiliations:** ^1^Division of Gastroenterology and Hepatology, Suining Central Hospital, Suining, Sichuan, China; ^2^Department of Radiology, Suining Central Hospital, Suining, Sichuan, China; ^3^Department of Radiology, Lixian People's Hospital, Aba, Sichuan, China

**Keywords:** foreign bodies, toothpick, pseudoaneurysm, gastrointestinal perforation, gastrointestinal bleeding, small intestine, iliac artery

## Abstract

Gastrointestinal (GI) bleeding is a common clinical condition that can be caused by a variety of reasons. Bleeding can occur anywhere in the GI tract, and it usually presents as vomiting of blood, melena or black stools. We herein present a case of a 48-year-old man who was ultimately diagnosed with perforation of the lower ileum, pseudoaneurysm of the right common iliac artery, lower ileum-right common iliac artery fistula, and pelvic abscess caused by accidental ingestion of a toothpick. This case suggests that accidental ingestion of a toothpick may also be the cause of GI bleeding in some patients. For patients with unexplained GI bleeding, especially those with small bowel bleeding, a rational and combined use of gastroduodenoscopy, colonoscopy, unenhanced and contrast-enhanced abdominal CT can help detect the causes of GI bleeding and improve diagnostic accuracy.

## Introduction

Gastrointestinal (GI) bleeding is a common clinical condition that includes mainly upper and lower GI bleeding. Bleeding can occur anywhere in the GI tract, whereas bleeding from the small intestine is relatively uncommon, accounting for ~5–10% of all patients with GI bleeding (1). GI bleeding can be caused by a variety of reasons, with GI inflammation, polyps, tumors, vascular diseases, infections, and systemic diseases being the most common causes (2–4). However, foreign bodies in the GI tract are a rare cause of small intestine bleeding (5). In this case report, we present a rare case of a 48-year-old man who was ultimately diagnosed with perforation of the lower ileum, pseudoaneurysm of the right common iliac artery, lower ileum-right common iliac artery fistula, and pelvic abscess caused by inadvertent ingestion of a toothpick.

## Case presentation

A 48-year-old male was presented to our hospital during the Chinese Spring Festival period with the chief complaint of melena for 10 days. He underwent both gastroduodenoscopy and colonoscopy in the local hospital, but no definite bleeding site was identified. He received hemostatic therapy and discharged after his condition improved. One day ago, the patient presented with melena again, and then visited our hospital. He had received surgical treatment for appendicitis 20 years ago and had a 30-year history of alcohol consumption (about 150 g/day). He had no previous history of liver diseases, coagulation disorders, GI bleeding, taking non-steroidal anti-inflammatory drugs (NSAIDs) or familial diseases. On admission, his body temperature was normal, blood pressure was 94/44 mmHg, with a heart rate of 106 beats/min. Physical examination showed an anemic face with pale skin and palpebral conjunctiva, mild tenderness in the lower abdomen, and no rebound pain. Routine blood test demonstrated a red blood cell count of 1.63 × 10^12^/L, hemoglobin level of 51 g/L, platelet count of 151 × 10^9^/L, white blood cell count of 7.1 × 10^9^/L. Fecal occult blood tests were positive (+), the results of other laboratory tests were normal. The patient's condition improved after receiving hemostasis, acid suppression, gastric protection therapies, fluid replacement, and infusion of red blood cell suspension (400 mL).

After a day of admission, the patient suddenly had bright red blood in his stool (about 1,500 mL), his blood pressure had dropped to 80/40 mmHg, with a heart rate 115 of beats/min. Routine blood test was performed again and revealed a red blood cell count of 1.55 × 10^12^/L, hemoglobin of 45 g/L, platelet count of 129 × 10^9^/L, and white blood cell count of 4.6 × 10^9^/L. The patient's condition was stabilized (blood pressure of 102/56 mmHg and heart rate of 98 beats per minute) after volume expansion therapy, fluid replacement, and infusion of red blood cell suspension (400 mL) and frozen plasma (200 mL).

**Figure 1:**
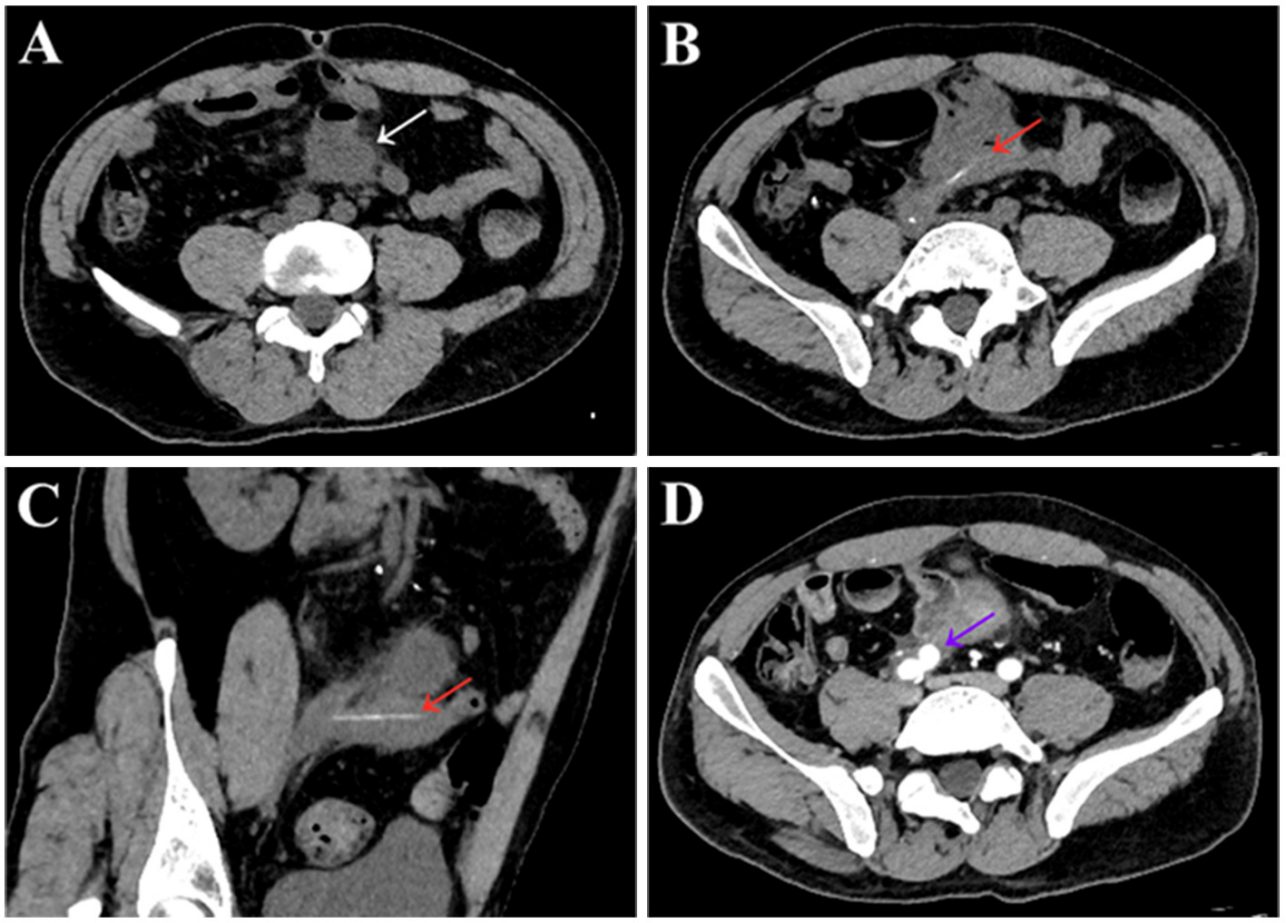
Unenhanced and contrast-enhanced abdominal CT images of a 48-year-old male patient. Unenhanced abdominal CT scans **(A–C)** showed encapsulated effusion in the middle part of the pelvic cavity, with accumulation of small amounts of gas [**(A)**, white arrow], and the lesion was ~4.5 cm × 5.5 cm, pelvic abscess formation was considered. A long strip-shaped slightly dense shadow was visible at the level below the pelvic abscess [**(B)**, red arrow], which was considered to be a pelvic foreign body. The length of the foreign body measured on the multiplanar reconstruction image was ~5.5 cm [**(C)**, red arrow]. Enhanced CT images in the arterial phase showed a pseudoaneurysm originating from right common iliac artery [**(D)**, purple arrow]. The size of the pseudoaneurysms was ~1.5 cm × 1.7 cm, with an entry tear of ~6 mm.

**Figure 2:**
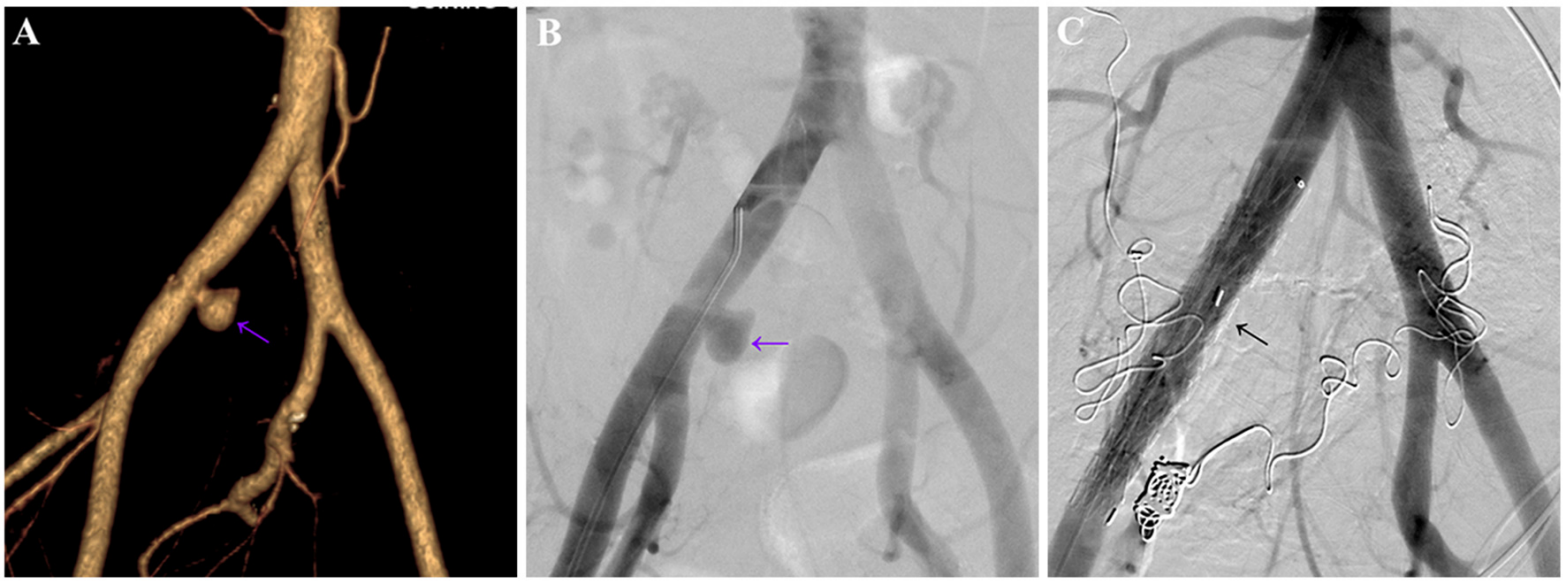
Volume-rendered, arterial-phase, contrast-enhanced CT image **(A)** and digital subtraction angiography (DSA) **(B)** of a 48-year-old male patient revealed a pseudoaneurysm originating from the right common iliac artery (purple arrow), the size of the pseudoaneurysm was ~1.8 cm × 2.1 cm, with an entry tear of ~6 mm. **(C)** DSA re-examination after stent placement showed complete disappearance of the pseudoaneurysm (black arrow).

To further clarify the etiology of the bleeding, unenhanced and contrast-enhanced abdominal computed tomography (CT) were performed. Unenhanced abdominal CT revealed encapsulated effusion in the middle part of the pelvic cavity, pelvic abscess was considered (Figure 1A, white arrow), and a long strip-shaped foreign body was visible below the level of the pelvic abscess (Figures 1B, C, red arrow). Contrast-enhanced CT image revealed a pseudoaneurysm originating from right common iliac artery (Figure 1D, purple arrow). Volume-rendered arterial phase CT image and digital subtraction angiography (DSA) confirmed the presence of a pseudoaneurysm originating from the right common iliac artery (Figures 2A, B, purple arrow).

**Figure 3:**
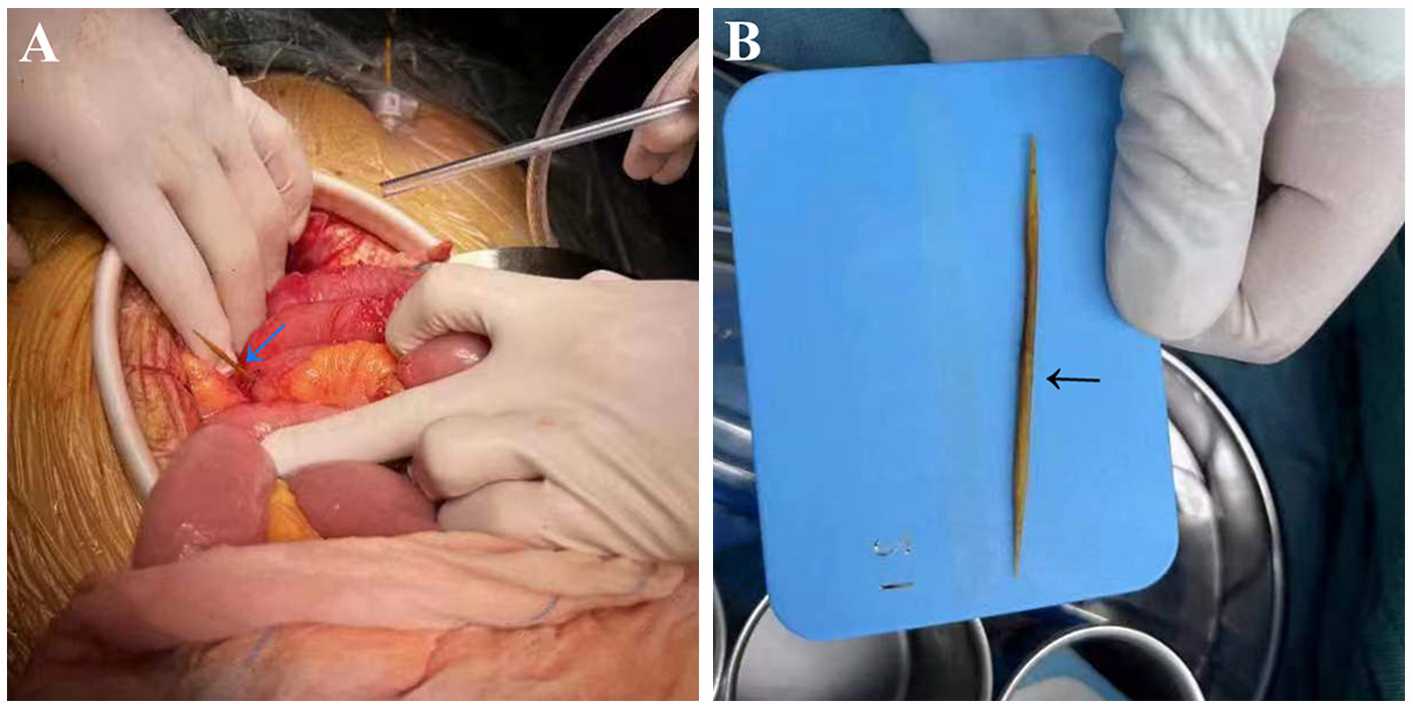
The foreign body was confirmed to be a toothpick (arrow) during exploratory laparotomy **(A)** and removed after surgery **(B)**.

**Figure 4:**
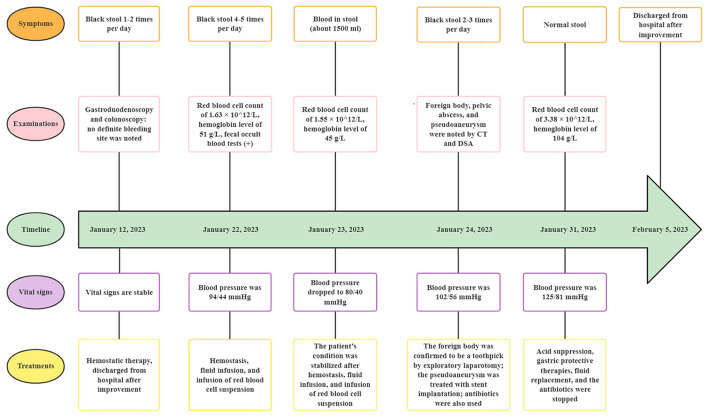
Medical timeline of the patient with small bowel bleeding. CT, computed tomography; DSA, digital subtraction angiography.

The patient underwent exploratory laparotomy, and the foreign body was finally confirmed to be a toothpick (Figures 3A, B). Since the toothpick had caused multiple perforations (5 perforation sites) in the lower ileum, and severe inflammatory edema occurred at the sites of perforation, with localized and encapsulated abscess formation, the toothpick was removed, and a portion of the ileum (~30 cm in length) was resected. Subsequently, the patient underwent stent placement for the treatment of the right common iliac artery pseudoaneurysm, post-operative DSA showed complete disappearance of pseudoaneurysm (Figure 2C, black arrow). Due to the presence of intra-abdominal infection, the patient received anti-infection therapy with imipenem cilastatin sodium after surgery (250 mg every 6 h for a week), and he recovered well after surgery without further GI bleeding. Medical timeline of the patient is shown in Figure 4.

Our patient reported the following about his disease: “During my hospitalization, I was concerned about being diagnosed with a gastrointestinal tumor. Fortunately, it was found to be a toothpick in my small bowel. I can't recall when and how the toothpick was swallowed into my small bowel. However, I am now in good health. Thank you to the doctors and nurses who have taken care of me and helped me.”

## Discussion

Foreign body ingestion is a common clinical phenomenon, most foreign bodies could pass through the GI tract smoothly, and can be ultimately expelled through the anus, while a few foreign bodies may be retained in the GI tract, causing inflammation, perforation, bleeding, obstruction, and possible damage to adjacent organs (6–9). Herein, we report a rare case of a 48-year-old man who was ultimately diagnosed with perforation of the lower ileum, right common iliac artery pseudoaneurysm, lower ileum-right common iliac artery fistula, and pelvic abscess caused by inadvertent ingestion of a toothpick. The patient's primary complaint was melena, so he underwent both gastroduodenoscopy and colonoscopy, but no definite bleeding site was identified. After unenhanced and contrast-enhanced abdominal CT was performed, accidental ingestion of a toothpick was confirmed to be the cause of GI bleeding.

**Figure 5:**
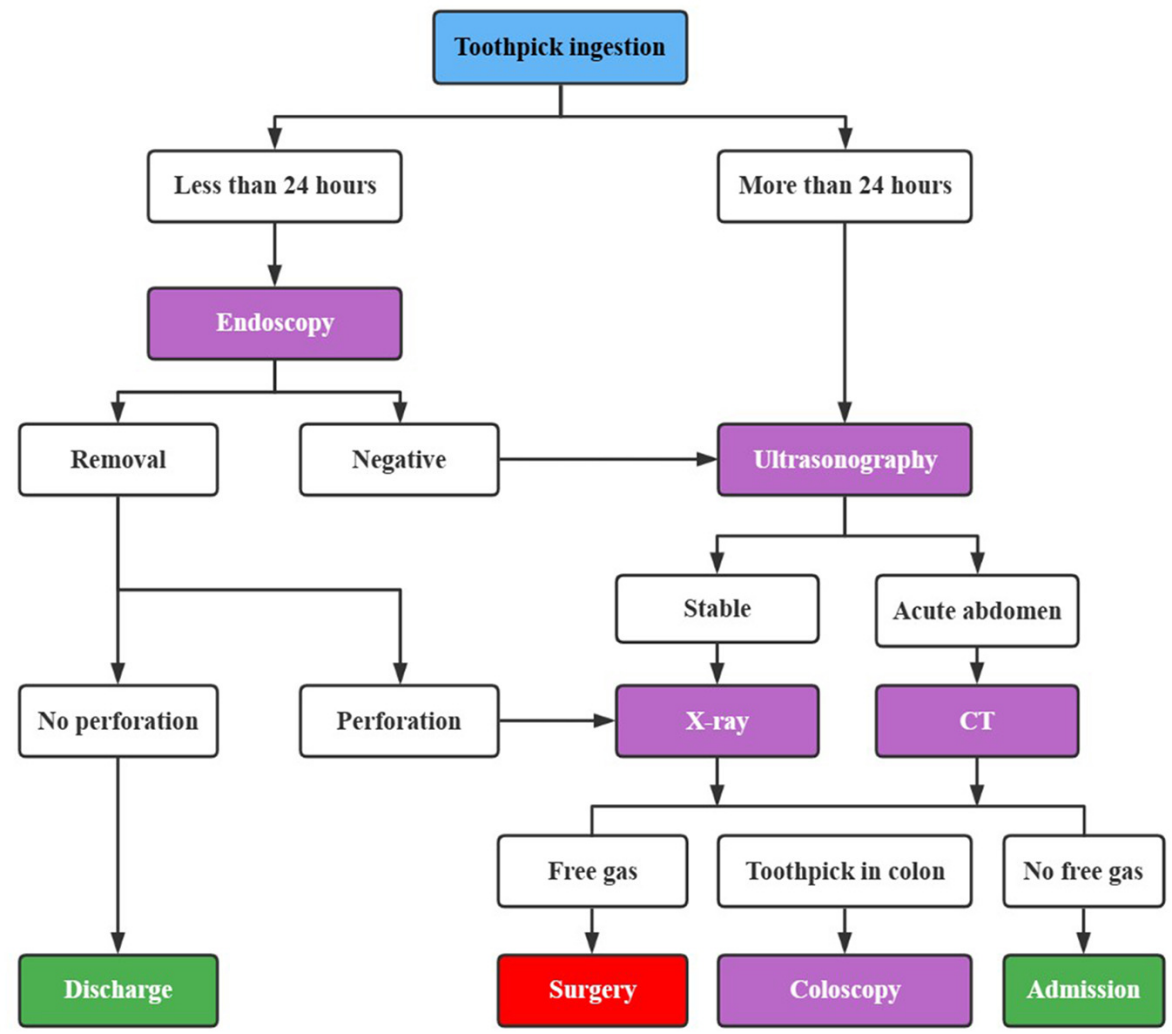
Algorithm for the management of toothpick ingestion. CT, computed tomography.

Steinbach et al. (9) proposed a diagnostic and therapeutic algorithm for the management of toothpick ingestion (Figure 5). In this algorithm, endoscopy was recommended as the most effective imaging technique for identifying and removing toothpicks after detection. If it does not work, or if toothpicks are ingested for more than 24 h, ultrasonography (US) is required. If the toothpicks are not detected by US, further diagnostic imaging would be needed. For patients with stable clinical conditions, an abdominal plain film radiography may be performed to eliminate free gas in the abdomen. While for patients with an acute abdomen, they would be examined with both unenhanced and contrast-enhanced abdominal CT.

We conducted a literature search on the PubMed (Medline) and Web of Science databases using the keywords “Pseudoaneurysm” and “Toothpick” on March 8, 2023, only 2 related published literatures were identified (10, 11). Nevertheless, the case we described differs from the above-mentioned two published studies in the following aspects. First, in this case, the presence of toothpick foreign body was mainly detected by abdominal CT; second, negative results were noted after the patient underwent first gastroduodenoscopy and colonoscopy; third, in this case, the ingested toothpick mainly caused ileal perforation and pseudoaneurysm of the right common iliac artery. Therefore, this case can be added to the world literature.

It is currently difficult to diagnose GI bleeding, and the diagnosis of small bowel bleeding due to foreign body ingestion is even more challenging, because most patients cannot recall a history of foreign body ingestion. Gastroduodenoscopy and colonoscopy are the methods of choice for the initial diagnosis and treatment of GI bleeding in patients (3). Due to the fact that the symptoms of small bowel bleeding are usually insidious with non-specific nature, and anatomically, the small intestine is a long tube, with complex arrangement and great mobility within the abdominal cavity, gastroduodenoscopy and colonoscopy are difficult to provide complete visualization of the entire small intestine, resulting in high rates of misdiagnosis and missed diagnosis of small bowel bleeding (12).

**Figure 6:**
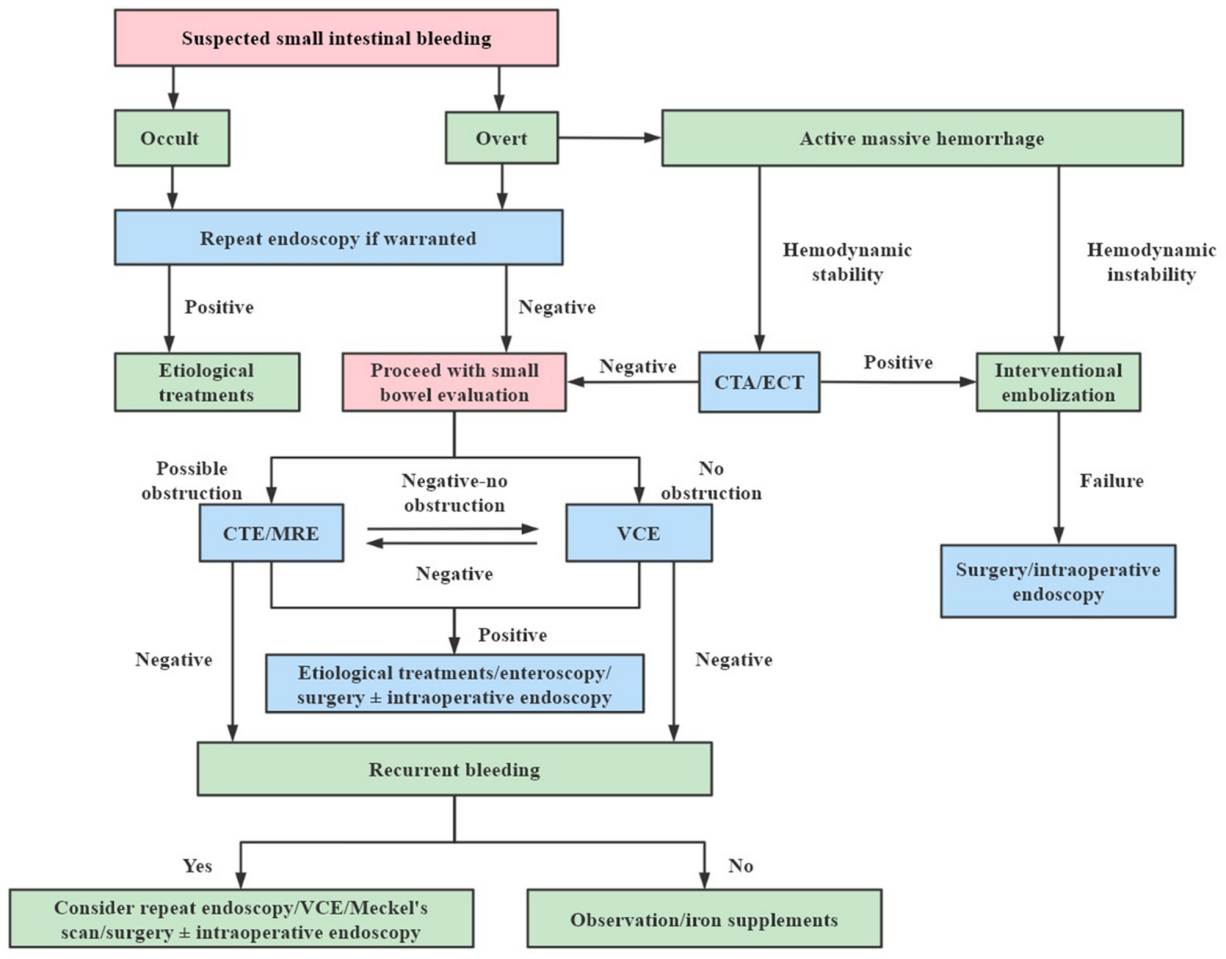
Algorithm for suspected small bowel bleeding. CTA, computed tomography angiography; ECT, emission computed tomography; CTE, computed tomography enterography; MRE, magnetic resonance enterography; VCE, video capsule endoscopy.

According to the algorithm for suspected small bowel bleeding proposed by a newly published guideline (13), if significant bleeding is not detected in patients after first gastroduodenoscopy and colonoscopy, a second gastroduodenoscopy and colonoscopy can be performed as needed (Figure 6). For patients with suspected small bowel bleeding, if they have active bleeding and are hemodynamically unstable, DSA should be performed as soon as possible, and embolization can be performed directly at the same time as the diagnosis is made. If the patients has active bleeding and are hemodynamically stable, CT angiography (CTA) or emission CT (ECT) is recommended to identify the site of bleeding. Video capsule endoscopy (VCE) is the first choice for patients with suspected small bowel bleeding who have stable vital signs, achieving a diagnostic rate of 38–87% (14). CT enterography (CTE) and magnetic resonance enterography (MRE) are the first-line methods for the detection of small bowel bleeding, which can be used in patients with a negative VCE or those who had contraindication to VCE and cannot undergo VCE (1, 15).

Therefore, for patients with unexplained GI bleeding, when the sites and causes of bleeding are not clearly identified during gastroduodenoscopy and colonoscopy, rational and combined use of VCE, enteroscopy, unenhanced and contrast-enhanced abdominal CT, CTE, MRE, CTA, DSA, and radionuclide methods should be considered by clinicians, and exploratory laparotomy can also be performed if necessary (16). Unenhanced and contrast-enhanced abdominal CT has the advantages of rapidity, convenience, not requiring intestinal preparation, and using various post-processing techniques like multiplanar reformation and volume rendering, which can rapidly detect GI bleeding and extraintestinal structural abnormalities, and can be used as an important adjunct in the diagnostic evaluation of GI bleeding, especially small bowel bleeding (17).

In our case, the patient was not identified the exact site and etiology of GI bleeding with the initial gastroduodenoscopy and colonoscopy examinations. However, in the subsequent CT examination, it was found to be a foreign body in the patient's small bowel. This indicates that a rational and combined application of gastroduodenoscopy, colonoscopy, and CT can help detect the causes of GI bleeding and improve diagnostic accuracy.

In our patient, a portion of the ileum (~30 cm in length) was removed. This was based on the reasons that the toothpick had caused multiple perforations (5 perforation sites) in the lower ileum, and severe inflammatory edema occurred at the sites of perforation, with localized and encapsulated abscess formation. The patient also underwent a stent placement procedure to treat the right common iliac artery pseudoaneurysm. This treatment method was consistent with our previous study (18). After the abdominal surgery, the patient received anti-infection therapy due to the presence of intra-abdominal infection.

In conclusion, this case suggests that accidental ingestion of a toothpick may also be the cause of GI bleeding in some patients. For patients with unexplained GI bleeding, especially those with small bowel bleeding, a rational and combined application of gastroduodenoscopy, colonoscopy, unenhanced and contrast-enhanced abdominal CT can help detect the causes of GI bleeding and improve diagnostic accuracy. After an accurate diagnosis, appropriate management can be performed.

## Data availability statement

The raw data supporting the conclusions of this article will be made available by the authors, without undue reservation.

## Ethics statement

The study was approved by the Institutional Ethics Committee of the Suining Central Hospital. Written informed consent was obtained from the participant/patient(s) for the publication of this case report.

## Author contributions

YX, JY, and GY participated in the study design and writing of the manuscript. GY, LF, YL, and SH participated in clinical data collection and analysis and carried out the interpretation of the CT images. GY, RL, GT, and BF revised the paper critically for intellectual content. All authors read and approved the final manuscript.
